# High pressure endoscopic irrigation: impact on renal histology

**DOI:** 10.1590/S1677-5538.IBJU.2020.0248

**Published:** 2021-02-03

**Authors:** Christopher Loftus, Michael Byrne, Manoj Monga

**Affiliations:** 1 University of Washington Medical Center Department of Urology Seattle WA United States Department of Urology, University of Washington Medical Center, Seattle, WA, United States; 2 Urology Richmond Virginia United States Urology, Richmond, Virginia, United States; 3 Glickman Urological and Kidney Institute Cleveland Clinic Foundation Cleveland Ohio United States Glickman Urological and Kidney Institute Cleveland Clinic Foundation - Urology, Cleveland, Ohio, United States

**Keywords:** Ureteroscopy, Calculi, Histology

## Abstract

**Purpose:**

High intra-renal pressures during flexible ureteroscopy have been associated with adverse renal tissue changes as well as pyelovenous backflow. Our objective was to investigate the effect of various intra-renal pressures on histologic changes and fluid extravasation during simulated ureteroscopy.

**Materials and Methods:**

Twenty-four juvenile pig kidneys with intact ureters were cannulated with an Olympus flexible ureteroscope with and without a ureteral access sheath and subjected to India ink-infused saline irrigation for 30 minutes at constant pressures ranging from sphygmomanometer settings of 50mm, 100mm and 200mmHg. Renal tissue samples were collected, processed and stained, and were evaluated by a blinded pathologist for depth of ink penetration into renal parenchyma as a percentage of total parenchymal thickness from urothelium to renal capsule.

**Results:**

The mean percentage of tissue penetration for kidneys with ink present in the cortical tubules at sphygmomanometer pressure settings of 50, 100, and 200mm Hg without a ureteral access sheath was 33.1, 31.0 and 99.3%, respectively and with ureteral access sheath was 0, 0 and 18.8%, respectively. Overall, kidneys with an access sheath demonstrated a smaller mean tissue penetration among all pressure compared to kidneys without a sheath (6.3% vs. 54.5%, p=0.0354). Of kidneys with sheath placement, 11% demonstrated any ink compared to 56% of kidneys without sheath placement.

**Conclusions:**

Pressurized endoscopic irrigation leads to significant extravasation of fluid into the renal parenchyma. Higher intra-renal pressures were associated with increased penetration of irrigant during ureteroscopy in an ex-vivo porcine model.

## INTRODUCTION

High intra-renal pressures may occur during ureteroscopy, percutaneous nephrolithotomy, and from hydronephrosis in an obstructed system and may result in the phenomenon of pyelovenous backflow by which there is communication of urine between renal fornices and renal veins (1–3). Intra-renal pressures that exceed 20-40mm Hg have been shown to result in pyelovenous backflow (4, 5). During endoscopic procedures in which heavy manual irrigation is used, intra-renal pressures can be quite high and even exceed 400mm Hg (6). In percutaneous nephrolithotomy it has been demonstrated that pyelovenous backflow due to high intra-renal pressure is associated with post-operative fever (5, 7). One study showed that after ureteroscopy, the rate of systemic inflammatory response syndrome (SIRS) may be around 8% and that when a ureteral access sheath (UAS) is used, larger diameters may be associated with lower rates of SIRS (8). Auge et al. studied intra-renal pressures during flexible ureteroscopy with a pressure transducer through a percutaneous nephrostomy tube and found that mean intra-renal pressure with a ureteroscope in the renal pelvis decreased by more than half when a UAS was in place (94 vs. 41mm Hg) (9). Furthermore, a larger diameter UAS has been shown to decrease intra-renal pressures and improve irrigation (10).

The goal of this study was to evaluate how irrigation pressures during ex-vivo ureteroscopy affect the depth of tissue penetration of collecting system irrigant on histology using an ink marker in a porcine renal system. We hypothesized that higher intra-renal pressures would be associated with increased depth of ink penetration and that use of UAS would decrease both intra-renal pressure and the degree of tissue penetration. The purpose of these experiments was to provide foundational framework and proof of concept for future studies which may choose to use these methods for investigating pyelovenous back-flow and extravasation of fluid from the renal pelvis.

## MATERIALS AND METHODS

Kidneys with intact ureters were taken from same-day sacrificed FDA grade pigs. The pigs were between the ages of 5 and 6 months old with a median weight of 73kg and were male gender. All male pigs had been castrated within the first 5 days of life. Renal systems were obtained freshly from a nearby slaughterhouse. Experiments were conducted within 6 hours post-mortem. Use of ex-vivo porcine renal systems has been shown in other experiments of simulate ureteroscopy (11).

To evaluate the effect of intra-renal pressure on fluid extravasation into kidney tissue, we set up experiments in which a ureteroscope was advanced retrograde into the ureter-kidney model and irrigated fluid to create pressure. Kidneys were divided into four pressure groups at which the external pressure of irrigant would be held constant: 50mm Hg, 100mm Hg, 200mm Hg, and no additional pressure. For each pressure setting, three experiments were performed on kidneys using a sheath and three experiments were performed on kidneys without a sheath.

A separate kidney was used for each experiment. In total 24 kidney were used- three for each sheath or no-sheath group within each pressure group.

To begin, an 8.4F flexible ureteroscope (Olympus URF Type P5, Olympus, Center Valley, PA) was inserted into ureters either with or without a ureteral access sheath. For experiments with sheaths, Flexor (Cook Medical, Bloomington, IN) access sheaths 35cm in length with 12/14F inner and outer lumen diameters were used. In such experiments, the sheath was advanced to the uretero-pelvic junction (UPJ) and secured in place with a loose circumferential 1-0 silk tie around the distal ureter to prevent the sheaths from slipping down to the distal ureter and to maintain their location. In the experiments when a sheath was not used, the ureter was similarly secured to the ureteroscope with the tip within the renal pelvis. For these experiments, the intention was not to simulate ureteroscopy (no manipulation of the ureteroscope was performed), but rather to reliably generate consistent intra-renal pressures. Normal saline was mixed with black India ink to create a 1.0% solution. The irrigant was attached directly to the ureteroscope 3.6F working channel. The pressure was generated using a sphygmomanometer wrapped around a new 3L bag of normal saline-ink solution. The sphygmomanometer was continually adjusted to hold at constant pressures of 50, 100, and 200mm Hg. For control kidneys at no additional pressure, 3L irrigant bags were used with no pressure applied but had flow due to the wall tension of the saline bag laid horizontal and level with the scope channel. Once the flow was opened inside the kidney, pressures were maintained for 30 minutes in each experiment. Intra-renal pelvic pressure was continuously monitored using General Electric CardioLab^®^ using a 5Fr catheter positioned at the ureteropelvic junction, placed retrograde alongside the ureteroscope.

After 30 minutes of irrigant flow, the kidneys were bivalved and a full thickness tissue sample was taken from the upper pole and the lower pole extending from the urothelium to the renal capsule. Samples were carefully taken so that they were uniform across experiments. Samples were fixed in 10% formalin solution for 7 days. After processing, samples were paraffin embedded, sectioned in 8 micrometer slices and stained with hematoxylin and eosin. The samples were then randomly assigned a study identification code and sent to blinded uropathologist who assessed for the presence and location of ink. The depth of penetration was determined as the maximum distance from the urothelium toward the renal capsule and the results were expressed at percentage of travel from urothelium to renal capsule. The depths were represented as a percentage of the total thickness of the parenchyma. Tubules were assessed for the presence of damage. Statistical analysis was performed using Fisher exact test for proportions and Wilcoxon signed-rank test for means.

## RESULTS

**Figure 1:**
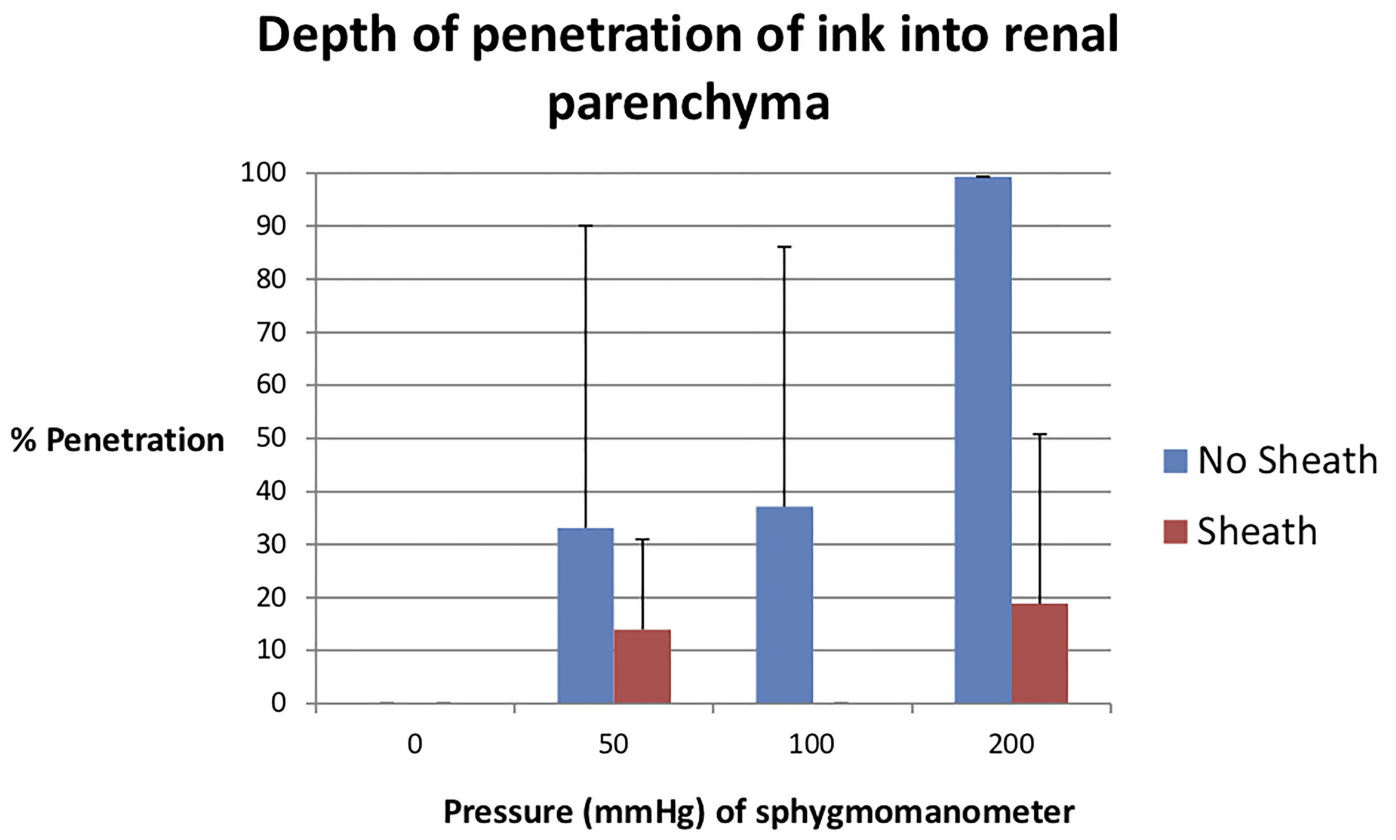
Measurements of depth of ink penetration for different pressure settings on sphygmomanometer. Data are shown as the percentage of penetration calculated as maximum distance of ink perfusion into tubules from urothelium divided by total distance from urothelium to renal capsule. No ink had measurable tissue penetration at irrigant pressure 0mm Hg and was not present when sheath was used at irrigant pressure of 50 and 100mg Hg.

In total, 24 kidneys were used with a mean individual renal weight of 160.3g (SD 21.5g). The observed intra-renal pressures were higher when a sheath was not used compared to when a sheath was employed at all irrigant sphygmomanometer pressure settings (p <0.001 for comparisons at each pressure setting) (Table-1). The control kidneys which did not receive pressurized irrigation demonstrated no tissue penetration of the ink. The mean percentage depth of tissue penetration of the ink into the cortical tubules from the urothelium to the renal capsule at sphygmomanometer pressures of 50, 100 and 200mm Hg without a UAS was 33.1, 31.0 and 99.3%, respectively and with a UAS was 14.0, 0 and 18.8%, respectively (Figure-1). At a sphygmomanometer pressure of 200mm Hg, sheath placement had significantly less penetration than without sheath placement p=0.046.

**Table 1:** Observed intra-renal pressures during various irrigant pressures with and without a sheath.

	No Sheath	With Sheath	p-value
50mm Hg	30.3 (SD 19.5)	1.2 (SD 1.6)	<0.001*
100mmg Hg	77.6 (SD 8.7)	4.7 (SD 0.63)	<0.001*
200mm Hg	123.8 (SD 18.9)	7.5 (SD 1.3)	<0.001*

Pressures expressed as mean pressure with standard deviations (SD). Comparisons were made between groups with and without sheaths,

* p-value <0.05 was considered significant.

**Figure 2:**
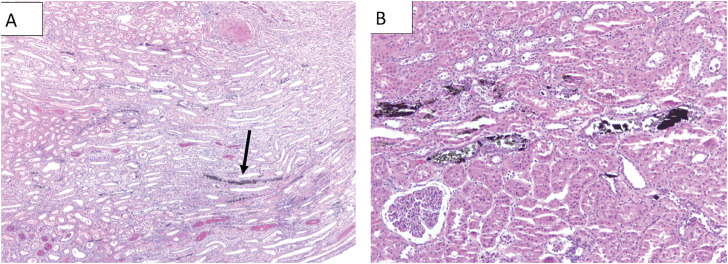
Renal parenchyma section with hematoxylin and eosin stain demonstrating ink perfusion into renal tubules. This kidney was in the group with sphygmomanometer setting at 200mm Hg without a sheath. 2A: Low power examination (100x) of renal parenchyma section, the arrow points to a longitudinally cut medullary tubules. 2B: High magnification (200x) of renal parenchyma section showing tubules with damage, sloughing of tubular epithelium within lumen, and ink spilling outside tubules.

Overall, kidneys with an access sheath demonstrated a smaller mean tissue penetration among all pressures compared to kidneys without a sheath (6.3% vs. 54.5%, p=0.035). The proportion of kidneys with the presence of any ink into the cortical tubules, at pressures of 50, 100 and 200 mmHg without a UAS was 0.33, 0.33 and 1.00, respectively and with a UAS was 0, 0 and 0.33, respectively. Examples of tissue perfusion are shown in Figure-2. Four of the five kidneys demonstrating significant tubular damage involving the medulla with detachment or dislodgement of tubular epithelium did not have sheaths.

## DISCUSSION

In this study, we described methods in which fluid extravasation into renal parenchyma can be measured using an ink mixture. We demonstrate that higher intra-renal pressures are associated with an increased distanced traveled of ink into renal parenchyma. This methodology may provide the foundation for future experiments aiming to measure the effects of endourologic procedures and their associated pressures on irrigant extravasation and pyelovenous backflow. This studied was designed not to replicate true ureteroscopic procedures in humans, but to provide a framework for future studies to employ such methods.

The importance of intra-renal pressures during endoscopic procedures has been highlighted by studies indicating that the degree of pyelovenous backflow of irrigation fluid and renal tissue damage is likely dependent with intra-renal pressure. A study by Schwalb et al. found that in mini-pig kidneys, intra-renal pressures could reach a maximum height of 439mm Hg during ureteropyeloscopy (12). In that study there were more histologic changes in kidneys exposed to high pressures compared to low pressures: acutely, high pressure kidneys demonstrated vacuolization and degeneration of tubules while subacutely high pressure kidneys had evidence of tubule scarring. However, it is unclear what the long-term clinical significance of these tissue changes may be. Studies have assessed the outcomes of patients with mild to moderate renal impairment undergoing ureteroscopy and found that the procedure does not appear to be associated with long-term renal function impairment (13, 14). High intrarenal pressures have also been associated with infectious complications. In a study comparing mini-PCNL to regular PCNL, mini-PCNL procedures demonstrated higher intra-renal pressures and had higher rates of end-organ seeding with bacteria which originated in the kidney (15). Furthermore, sustained time at high intra-renal pressures have been associated with post-operative fever (8). These data suggest that high intra-renal pressures may lead to adverse outcomes in some cases.

Our study did not specifically measure bacterial movement during high intra-renal pressures and instead used ink as a marker. We believe that this technique of monitoring for ink perfusion could be used in experiments whose goal is to evaluate tissue intravasation of infected fluid or even tumor. Studies have speculated whether endoscopic procedures for diagnosis and management of upper-tract urothelial carcinoma may “seed” the upper tracts with additional cancer and cause tumor cells to implant into renal parenchyma, however endoscopic surveillance has not shown significant change in clinical outcomes (16, 17). Using the methods we demonstrate in our study, this topic could potentially be further studied in ex-vivo or in-vivo animal models using tumor cells and ink to visualize tissue penetration.

We conducted this study to demonstrate the impact of intra-renal pressure on degree of extravasation of irrigant fluid during endoscopic procedures. Our study demonstrated that in simulated ureteroscopy using a porcine model, high perfusion pressures are associated with a significant increase of India ink infiltration into renal tissue. We found a pressure dependent relationship for the ability of ink to penetrate both into and outside of tubules which was abolished with sheath placement. By allowing an outlet for irrigant fluid with the sheath, intra-renal pressures were lower at the same infusion pressures. These results lend further evidence to the growing literature that ureteral access sheaths may help protect renal tissue from damage and systemic infection. The experiments without a sheath included a tie around the ureter to prevent the ureteroscope from slipping distally and this almost certainly hindered fluid from traveling around the ureteroscope to escape. These methods were performed because our goal was to keep intra-renal pressures relatively constant. We did not intend to simulate true ureteroscopy. Other studies have demonstrated that antegrade leakage of fluid is important during ureteroscopy to help reduce pressures and promote irrigation (18). In our study, there may have been variable intra-renal pressures due to our attempt to maintain a constant pressure of the sphygmomanometer. We used experiments with and without an access sheath because prior studies have shown that using a sheath may decrease the intra-renal pressure during endoscopic procedures (19). It is interesting that some of the experiments at 100mm Hg pressure setting and even the lower pressure settings did not display extravasation of ink. This may be due to the washout process during tissue preparation for parafinization and may be a limitation of using ink as a marker. For subsequent experiments which may emerge with these methods, one could investigate the effect that pressure regulation has one fluid penetration. A study which retrospectively compared ureteroscopy with manual hand irrigation vs. gravity pressure bags showed that the manual irrigation cohort had higher rates of post-operative fever, SIRS and emergency department presentation (20). It could be that quick, transient changes in pressure are more likely to drive backflow of irrigant.

The histologic effects of ureteroscopy in human kidneys is not known. Our study used porcine kidneys which have been validated as suitable models of human kidneys (21, 22). This study has several limitations. We did not perform true simulated ureteroscopy as the scope was not manipulated during the procedures, there was no active lasering of stones, and the scope was secured to the ureter to allow for consistent intra-renal pressures. We also ran each experiment for only 30 minutes. Thus, the procedures in this study do not mirror real-life ureteroscopy in humans. However, damage of urothelium with laser or stone movement could potentially provide a mechanism for collecting system irrigant to extravasate into the renal parenchyma which we were not able to study. Additionally, due to difficulties with the ureteroscope slipping out of the floppy, acontractile ureter, we were required to place a silk tie gently around the distal ureter which likely increased intra renal pressures for all experimental pressures and decreased antegrade flow. It was not our goal to simulate a true human ureteroscopy, but rather demonstrate the effect that intra-renal pressures have on tissue penetration of irrigant and evaluate whether these effects were mitigated by access sheath placement. Furthermore, we attempted to collect uniform samples from the upper and lower poles of each kidney after completion of the experiment. The blinded uropathologist measured the distance from the urothelium to the tubule with ink penetration which was nearest the renal capsule. However, slight changes in how the samples were cut in sectioning could potentially change this maximal distance so percentages were used instead of raw distances. Future studies may evaluate tissue penetration in a more clinical scenario with true, in vivo flexible ureteroscopy in the porcine model. Alternative routes of backflow may also be assessed in future investigations. Here, black India ink was a surrogate to monitor extravasation of fluid but because the ink molecules can clump and get stuck in tubules, total migration of ink may underestimate the degree of intrarenal fluid penetration.

This study demonstrated that in ureteroscopy, a procedure known to have a large variance in intra-renal pressure (9), increasing irrigation pressure resulted in deeper tissue penetration of ink. Additionally, tissue penetration of ink was higher when a UAS was not used. With sheath use, there was no significant increase in tissue penetration. We conclude that the technique of using ink in settings of high intra-renal pressures is a reliable way to measure extravasation of fluid into renal tissue.

## CONCLUSIONS

The histological effects of ureteroscopy in human kidneys it not known. This study demonstrated that in a porcine model, higher pressures of saline irrigation in simulated ureteroscopy result in an increase in degree of tissue penetration by the intra-renal fluid. Future studies may use ink has a marker to monitor for extravasation of fluid in other clinical contexts.

## ABBREVIATIONS

UAS: ureteral access sheath
